# Approaches to Type 1 Diabetes Prevention by Intervention in Cytokine Immunoregulatory Circuits

**DOI:** 10.1155/EDR.2001.3

**Published:** 2001

**Authors:** Wilma L. Suarez-Pinzon, Alex Rabinovitch

**Affiliations:** 1 Department of Medicine University of Alberta 430 Heritage Medical Research Centre Alberta Edmonton T6G 2S2 Canada

## Abstract

Type 1 (insulin-dependent) diabetes mellitus, like other organ specific autoimmune diseases, results from a disorder of immunoregulation. T cells specific for pancreatic islet ß cell constituents (autoantigens) exist normally but are restrained by regulatory mechanisms (self-tolerant state). When regulation fails, ß cell-specific autoreactive T cells become activated and expand clonally. Current evidence indicates that islet ß cell-specific autoreactive T cells belong to a T helper 1 (Th1) subset, and these Th1 cells and their characteristic cytokine products, IFNγ and IL-2, are believed to cause islet inflammation (insulitis) and ß cell destruction. Immune-mediated destruction of ß cells precedes hyperglycemia and clinical symptoms by many years because these become apparent only when most of the insulin-secreting ß cells have been destroyed. Therefore, several approaches are being tested or are under consideration for clinical trials to prevent or arrest complete autoimmune destruction of islet ß cells and insulin-dependent diabetes. Approaches that attempt to correct underlying immunoregulatory defects in autoimmune diabetes include interventions aimed at i) deleting ß cell autoreactive Th1 cells and cytokines (IFNγ and IL-2) and/or ii) increasing regulatory Th2 cells and/or Th3 cells and their cytokine products (IL-4, IL-10 and TGFßI).


### Approaches to Type 1 Diabetes Prevention by Intervention in Cytokine Immunoregulatory Circuits

WILMA L. SUAREZ-PINZON AND ALEX RABINOVITCH*

*Department of Medicine, University of Alberta, 430 Heritage Medical Research Centre, Edmonton, Alberta, T6G 2S2, Canada
E-mail: alex.rabinovitch@ualberta.ca

### Abstract

Type 1 (insulin-dependent) diabetes mellitus, like other organ specific autoimmune diseases, results from a disorder of immunoregulation. T cells specific for pancreatic islet ß cell constituents (autoantigens) exist normally but are restrained by regulatory mechanisms (self-tolerant state). When regulation fails, ß cell-specific autoreactive T cells become activated and expand clonally. Current evidence indicates that islet ß cell-specific autoreactive T cells belong to a T helper 1 (Th1) subset, and these Th1 cells and their characteristic cytokine products, IFNγ and IL-2, are believed to cause islet inflammation (insulitis) and ß cell destruction. Immune-mediated destruction of ß cells precedes hyperglycemia and clinical symptoms by many years because these become apparent only when most of the insulin-secreting ß cells have been destroyed. Therefore, several approaches are being tested or are under consideration for clinical trials to prevent or arrest complete autoimmune destruction of islet ß cells and insulin-dependent diabetes. Approaches that attempt to correct underlying immunoregulatory defects in autoimmune diabetes include interventions aimed at i) deleting ß cell autoreactive Th1 cells and cytokines (IFNγ and IL-2) and/or ii) increasing regulatory Th2 cells and/or Th3 cells and their cytokine products (IL-4, IL-10 and TGFß1).

Key Words: Type 1 Diabetes, Autoimmunity, Immunoregulation, Cytokines

### Type 1 Diabetes Viewed as a Disorder of Immunoregulation

Type 1 diabetes mellitus results from selective destruction of the insulin-producing ß cells in the pancreatic islets of Langerhans. The current concept is that pancreatic islet ß cells are destroyed by an *autoimmune response* mediated by T lymphocytes (T cells) that react specifically to one or more ß cell proteins (autoantigens).[1] Although it has not

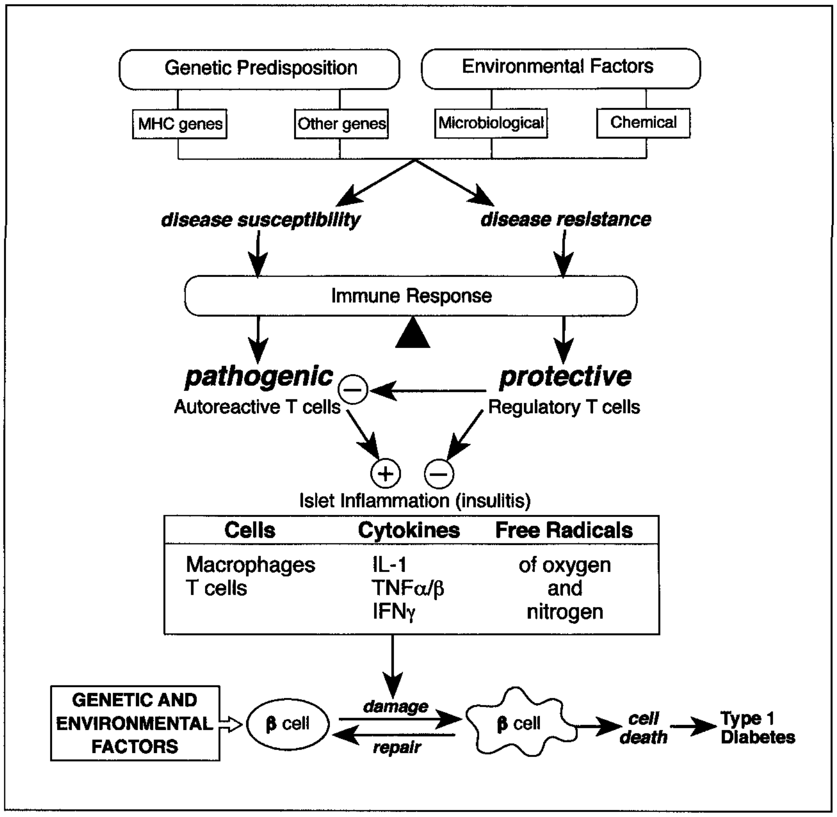

FIGURE 1
A current formulation of the pathogenesis of type 1 diabetes. Genetic and environmental factors interact and confer either susceptibility or resistance to disease, depending on the gene/allele possessed by the individual and the environmental agent to which that individual is exposed. Disease susceptibility leads to a pathogenic immune response whereas disease resistance leads to a protective immune response. The pathogenic immune response is believed to be mediated by T lymphocytes (T cells) that are reactive to islet ß cell self-antigen(s) (autoreactive T cells), whereas a protective immune response may be mediated by T cells that suppress the autoreactive T cells (regulatory T cells). Dominance of the pathogenic immune response would lead to islet inflammation (insulitis). This is characterized by infiltration of the islet by macrophages and T cells that are cytotoxic, both directly and indirectly by producing cytokines (e.g., IL-1, TNFα, TNFß, and IFNγ) and free radicals that damage ß cells. Genetic and environmental factors may also directly increase or decrease the ability of ß cells to repair damage and prevent irreversible ß cell death, insulinopenia, and diabetes. (Reproduced from Rabinovitch A. and Skyler JS. Prevention of type 1 diabetes 1998;82:739, with permission of W.B. Saunders Co.)

been excluded that a primary ß cell lesion, intrinsic or acquired (possibly viral or chemical), might be involved in *initiating* an autoimmune response,[2] it is clear that, once established, an immune response is the cause of ß cell destruction. For example, diabetes transfer studies have demonstrated that bone marrow-derived cells from hosts with autoimmune diabetes can transfer ß cell destructive insulitis to nondiabetes-prone human, mouse, or rat pancreas, thereby indicating that an underlying abnormality in type 1 diabetes resides in the immune system.[3-8]

The autoimmune response to islet ß cells is thought to occur in persons who possess certain susceptibility alleles and who lack other protective alleles of the major histocompatibility (MHC) gene complex, which regulates immune responses. In addition, non-MHC genes may contribute to the autoimmune response. The traditional concept is that environmental factors (e.g., microbial, chemical, dietary) may trigger an autoimmune response against ß cells in a genetically diabetes-prone individual. Studies in animal models with spontaneous autoimmune diabetes, however, have revealed that environmental factors (particularly microbial agents) may either *promote or protect against* diabetes development.[9] Therefore, the current concept being explored is that both genetic and environmental inputs may be either pathogenic (i.e., diabetes-promoting) or protective against type 1 diabetes, and that disease appearance is influenced by the net effects of genetic and environmental factors on immune responses. According to this concept, type 1 diabetes, like other organ-specific autoimmune diseases, results from a disorder of immunoregulation.[1] This posits that T cells specific for islet ß cell molecules (i.e., autoantigens) exist normally but are restrained by immunoregulatory mechanisms (the self-tolerant state), and that type 1 diabetes develops when one or another immunoregulatory mechanism (e.g., regulatory T cells) fails, allowing ß cell-autoreactive T cells to become activated, expand clonally, and entrain a cascade of immune and inflammatory processes in the islets, culminating in ß cell destruction (Fig. 1).

Although it is not known what may trigger loss of self-tolerance to islet antigens in type 1 diabetes, it appears that defective immunoregulatory (suppressor) mechanisms allow the autoimmune state to progress to a pathological level and cause ß cell destruction. There is now abundant evidence that suppressor cell defects may contribute to diabetes development in rodent models of type 1 diabetes. In the nonobese diabetic (NOD) mouse, diabetes onset is accelerated by thymectomy performed at 3 weeks of age[10] and by administration of cyclophosphamide,[11,12] a drug known for its selective effects on suppressor T cells. Diabetes transfer is obtained only in immunodeficient recipients, that is, neonates[13] and adults that have been sublethally irradiated[14] or thymectomized and treated with a monoclonal antibody to CD4+ T cells.[15] One can prevent diabetes transfer by spleen cells from diabetic mice by preinfusion of CD4+ spleen cells from nondiabetic syngeneic mice.[16] CD4+ and CD8+ suppressor clones have been reported,[17-19] as has the production of a suppressor factor.[19] Treatment of young NOD mice with an anti-MHC class II monoclonal antibody protects them from diabetes, and this protection is transferable to non-antibody-treated mice by infusion of CD4+ T cells from protected mice.[20] In the Biobreeding (BB) rat, diabetes is accelerated by the administration of a monoclonal antibody to RT6.1+ T cells[21] and prevented by transfusion of lymphoid cells from diabetes-resistant BB rats.[22] Finally, the mechanisms by which islet autoreactive T cells may be suppressed are unknown; however recent studies have pointed to cytokines as important immunoregulatory molecules.

### Immune Responses: Roles of Cytokines

Cytokines are peptide molecules synthesized and secreted by activated lymphocytes (lymphokines), macrophages/monocytes (monokines) and cells outside the immune system (e.g., endothelial cells, bone marrow stromal cells, and fibroblasts). Cytokines are used mainly by immune system cells to communicate with each other and to control local and systemic events of immune and inflammatory responses. More than 30 immunologically active cytokines exist and are generally grouped as interleukins (ILs), interferons (IFNs), tumor necrosis factors (TNFs), and colony-stimulation factors (CSFs).[23] Both the production of cytokines by cells and the actions of cytokines on cells are complex: A single cell can produce several different cytokines, a given cytokine can be produced by several different cell types, and a given cytokine can act on one or more cell types. Also, cytokine actions are usually local: It can act i) between two cells that are conjugated to one another, ii) on neighboring cells (paracrine), and iii) on the cell that secretes the cytokine (autocrine). In some cases (notably the macrophage-derived inflammatory cytokines, such as IL-1, IL-6, and TNFα) cytokines exert actions on distant organs (endocrine).

Interpretation of the actions of cytokines in general is complicated by the very nature of cytokine biology. First, large amounts of a cytokine are often produced when a cell is stimulated by an antigen, mitogen, or other cytokines (e.g., up to 2% of cell protein synthesis can be devoted to a single cytokine). Second, cytokine receptors have high affinities for their specific cytokine ligands, so most cytokines have very high specific activity. The consequences of these properties of cytokines and cytokine receptors is that one activated cell can produce enough cytokine to activate 1,000 - 10,000 other cells (i.e., a very small number of antigen-reactive cells can have widespread effects). Third, cytokine synthesis is regulated by the differentiation of cells into the various cytokine-secreting phenotypes and by the selective activation of different cell types to produce some or all of their characteristic set of cytokines.

Antigen-activated T cells are termed T helper (Th) cells because they help to mediate both cellular and humoral (antibody) immune responses. In 1986, Mosmann and colleagues,[24] started a conceptual revolution in immunology by dividing T helper (Th) cells into two populations with contrasting and crossregulating cytokine profiles. The Th1 and Th2 patterns of cytokine production were originally described among mouse CD4+ T cell clones[24,25] and later among human T cells.[26] Mouse Th1 cells produce IL-2, IFNγ, and TNFß (also termed lymphotoxin), whereas Th2 cells produce IL-4, IL-5, IL-6, IL-9, IL-10 and IL-13. Cytokine production by human Th1 and Th2 cells follows similar patterns, although the synthesis of IL-2, IL-6, IL-10 and IL-13 is not as tightly restricted to a single subset as in mouse T cells. Several other proteins are secreted both by Th1 and Th2 cells, including IL-3, TNFα, granulocyte-macrophage colony-stimulating factor (GM-CSF) and members of the chemokine families.[27] Th1 and Th2 responses are not the only cytokine patterns possible: T cells expressing cytokines of both patterns have been called Th0 cells[28] and those producing high amounts of transforming growth factor ß (TGFß) have been termed Th3.[29]

The functional significance of Th1 and Th2 cell subsets is that their distinct patterns of cytokine secretion lead to strikingly different T cell actions.[27,28,30-32] Th1 cells and their cytokine products (IL-2, IFNγ and TNFß) are the mediators in cell-mediated immunity (formerly termed delayed-type hypersensitivity). IFNγ and TNFß activate vascular endothelial cells to recruit circulating leukocytes into the tissues at the local site of antigen challenge, and they activate macrophages to eliminate the antigen-bearing cell. In addition, IL-2 and IFNγ activate i) cytotoxic T cells to destroy target cells expressing the appropriate MHC-associated antigen, and ii) natural killer (NK) cells to destroy target cells in an MHC-independent fashion. Thus, Th1 cytokines activate cellular immune responses.

In contrast, Th2 cytokines are much more effective stimulators of humoral immune responses, i.e., immunoglobulin (antibody) production, especially immunoglobulin E, by B cells. Furthermore, responses of Th1 and Th2 cells are mutually inhibitory. Thus, the Th1 cytokine IFNγ inhibits the production of the Th2 cytokines IL-4 and IL-10; these, in turn, inhibit Th1 cytokine production.

Protective responses to pathogens depend on activation of the appropriate Th subset accompanied by its characteristic set of immune effector functions. For example, human Th1 cells develop in response to intracellular bacteria and viruses, whereas Th2 cells develop in response to allergens and helminth components.[30] Th1 and Th2 cells play different roles not only in protection against exogenous offending agents, but also in immunopathology. Th1 cells are involved in contact dermatitis, organ-specific autoimmunity, and allograft rejection, whereas Th2 cells are responsible for initiation of the allergic cascade.[30]

Among signals that may orient the immune response in the direction of either a Th1 or a Th2 cell response, the macrophage-derived cytokines, IL-10[33] and IL-12[34] have been discovered to play important roles. IL-12 is a potent stimulant of Th1 cells and cytokines, notably IFNγ. Thus, IL-12 can initiate cell-mediated immunity. In contrast, IL-10 (derived from macrophages and Th2 cells) exerts anti-inflammatory effects by inhibiting production of IL-12 and other pro-inflammatory macrophage cytokines (e.g., IL-1, IL-6, IL-8, TNFα), by increasing macrophage production of IL-1 receptor antagonist, and by inhibiting the generation of oxygen and nitrogen free radicals by macrophages. In addition, IL-10 may favor Th2 over Th1 cell differentiation and function by inhibiting expression of MHC class II molecules and the B7 accessory molecule on macrophages, a major costimulator of T cells.[35] The combination of IL-4 and IL-10 is particularly effective in inhibiting Th1 effector function (i.e., cell-mediated immunity) *in vivo*.[36]

Th1-like and Th2-like polarized cytokine secretion patterns have now been described for many different cell types: CD4, CD8 and γδ T cells, NK cells, B cells, dendritic cells, macrophages, mast cells and eosinophils.[37] In recognition of the fact that cytokine secretion patterns are not restricted to certain cell types, they are often described as type 1 and type 2 rather than Th1 and Th2. Thus a cytokine can be classified *on the basis of the response it evokes rather than on the cell type that produces it*.[38] Type 1 cytokines (IFNγ, IL-2, TNFß and IL-12) primarily stimulate cell-mediated immunity; type 2 cytokines (IL-4, IL-5, IL-6, IL-10 and IL-13) primarily induce humoral immunity and diminish cellular immunity; and the type 3 cytokine (TGFß) also diminishes cellular immunity. IL-1 (both α and ß isoforms) and TNF are primarily, but not exclusively macrophage-derived cytokines, and are generally referred to as proinflammatory cytokines.

### Autoimmune Diabetes: A Dominance of Th1 Over Th2 Cells?

There is now abundant evidence that autoreactive T cells are present in the normal immune system but are prevented from expressing their autoreactive potential by other regulatory (suppressor) T cells. For example, reconstitution of lymphopenic, prediabetic BB rats with the IL-4-producing $CD4^+$ $CD45RC^{low}$ subset of Th cells but not with the IL-2-producing $CD4^+$ $CD45RC^{high}$ Th subset protects against autoimmune diabetes.[39] In a different model, adult thymectomy combined with sublethal irradiation causes diabetes in a nonautoimmune diabetes-prone rat strain, and insulitis and autoimmune diabetes are completely prevented by injection of $CD4^+$ $CD45RC^{low}$ T cells that secrete IL-2 and IL-4, not IFNγ.[39,40] Diabetes can be adoptively transferred into neonatal NOD mice or immunocompromised NOD-*scid* by splenic cells from diabetic NOD mice, whereas splenic cells from young nondiabetic NOD mice can prevent diabetic splenic cells from adoptively transferring disease. Interestingly, both the pathogenic and protective functions of $CD4^+$ cells in the diabetic and nondiabetic NOD donor spleens were found to reside in a $CD45RB^{low}$ subset of $CD4^+$ T cells; however, the pathogenic cells had a significantly higher IFNγ/IL-4 production ratio than did the protective ones.[41] These findings support the concept that Th1 cells (IFNγ-producing) are pathogenic and Th2 cells (IL-4-producing) prevent diabetes development; however, diabetes transfer and prevention were observed using polyclonal populations of T cells, and the autoimmune response in type 1 diabetes is believed to be dependent on T cells specifically reactive to islet ß-cell autoantigens.

A variety of *islet-reactive* T cell lines and clones that either adoptively transfer diabetes or prevent against its development in NOD mice have been described, and some of these T cell lines/clones have been characterized in terms of their cytokine production profiles. In one study, $CD4^+$ T cells reactive to the islet autoantigen, glutamic acid decarboxylase (GAD), were reported to secrete IFNγ, TNFα, and TNFß, but not IL-4 in response to GAD antigen, and these cells adoptively transferred diabetes into NOD-*scid* mice.[42] Interestingly, several diabetes-preventive $CD4^+$ T cell clones were found to produce a variety of cytokines, including type 1 cytokines (IFNγ and TNFß), a type 2 cytokine (IL-10), and a type 3 cytokine (TGFß).[43-45] TGFß was implicated as the mediator of the diabetes-preventive effects of these islet-reactive CD4+ T cell clones.[44,45] In another study, CD4+ T cell lines that react to rat insulinoma cells and secrete either IFNγ or IL-4 were developed from spleens of diabetic NOD mice.[46] The IFNγ-secreting CD4+ T cells (Th1-type) adoptively transferred ß cell destructive insulitis and diabetes into neonatal NOD mice, whereas the IL-4- secreting CD4+ T cells (Th2-type) induced a nondestructive peri-islet insulitis.[46] Similarly, Th1 cells expressing a diabetogenic T cell receptor adoptively transferred ß cell destructive insulitis and diabetes in neonatal NOD mice, whereas Th2 cells expressing the same T cell receptor did not; however, the Th2 cells did not prevent the Th1 cells from transferring diabetes.[47] This suggests that Th2 cells *cannot* downregulate Th1 cells whose effector functions (e.g., type 1 cytokine production) are fully differentiated.

In contrast, a subset of natural killer thymocytes (NKT), TCRαß+CD4CD8, has recently been reported to prevent adoptive transfer of diabetes by diabetogenic NOD splenocytes, and protection was related to IL-4 and/or IL-10 production.[48] The protection provided by the NKT cells is believed to represent diabetes prevention by correction of an underlying deficiency of NKT cells[49,50] and IL-4 production[51] in NOD mice. In another recent study, a subset of TCRαß+CD4+CD62L+ thymocytes was reported to prevent adoptive transfer of diabetes by diabetogenic NOD splenocytes,[52] however, the cytokine-producing phenotype of these CD4+ regulatory T cells was not determined. Collectively, these studies have given rise to the concept that the autoimmune response in type 1 diabetes involves disturbances in immunoregulatory circuits manifested as a dominance of Th1 over Th2 cell function and cytokine production (Fig. 2).

According to the scheme depicted in Figure 2, certain ß cell protein(s) act as autoantigens after being processed by antigen-presenting cells (APCs), such as macrophages, dendritic cells, and B cells. APCs appear to play an important role in the initiation of insulitis. Thus, many studies indicate that macrophages and dendritic cells are the first cells to infiltrate pancreatic islets,[53-55] and inactivation of macrophages results in the near-complete prevention of insulitis and diabetes in both NOD mice and BB rats.[56,57] Recent studies have found that macrophages play an essential role in diabetes development in NOD mice by activating, largely through IL-12 secretion, Th1 cells and CD8+ cytotoxic T cells.[58,59] Also, recent studies have revealed that B cells clearly influence diabetes development in a manner that probably relates to their APC function, and lack of B cells prevents diabetes development.[60-62] The immunogenicity of a ß cell protein may depend upon the peptide fragment derived from processing by the APC,[63] the amino acid sequences of the MHC class II molecules that bind and present the ß cell peptide (antigen), and the precursor frequency of autoreactive T cells with T cell receptors to match the ß cell antigen-MHC complex.[64] Interestingly, both non-MHC genes[65] and MHC class II genes[66] have been reported to determine the polarity of the Th1/Th2 immune response in NOD mice.

In addition to the MHC-antigen complex interaction with T cell receptors, T cell activation by APCs involves costimulation through multiple ligand/receptor pairs, e.g., B7/CD28, CD40L/CD40, and ICAM-1/LFA-1.[67,68] There is evidence that APC-T cell interactions via these costimulatory molecules are involved in diabetes pathogenesis. For example, transgenic expression of the costimulator molecule, B7-1 (CD80) in islet ß cells has been shown to accelerate diabetes in NOD mice.[69] Also, NOD female mice did not develop diabetes when treated, at the onset of insulitis (2-4 weeks of age), with CTLA4 immunoglobulin (a soluble antagonist to CD28, the T cell receptor for the B7 ligand on APCs) or a monoclonal antibody specific for B7-2 (CD86).[70] In addition, anti-CD40L monoclonal antibody treatment of NOD female mice (3-4 weeks of age, but not greater than 9 weeks of age) completely prevented insulitis and diabetes.[71] Blockade of ICAM- 1 and LFA-1 by injection of monoclonal antibodies[72,73] or soluble forms of ICAM-1,[74] reduced insulitis and diabetes incidence in NOD mice, and treatments with the soluble forms of ICAM-1 were found to decrease IFNγ mRNA expression in the pancreas.[74]

The direction taken by the T cell response, in terms of Th phenotype, is largely regulated by cytokines. Thus, naive T cells are not precommitted to any particular Th phenotype; the Th phenotype varies with the cytokines in the microenvironment. The presence of IL-12, a macrophage and B cell product, favors Th1 cell differentiation, and anti-IL-12 antiserum blocks expression of the Th1 phenotype.[75] Indeed, administration of IL-12 to prediabetic NOD female mice was found to accelerate diabetes onset, and this was associated with i) enhanced IFNγ and decreased IL-4 production by islet-infiltrating lymphocytes, and ii) selective ß cell destruction.[76] IL-4, a Th2 and possibly a mast cell product,[77] favors Th2 cell differentiation, and anti-IL-4 monoclonal antibody promotes expression of a Th1 phenotype.[77,78] The results of Th1 cell activation are induction of IL- 2 and IFNγ production, inhibition of Th2 cytokine production, and activation of macrophages, cytotoxic T cells, and natural killer cells. These activated *effector* cells may be cytotoxic to islet ß

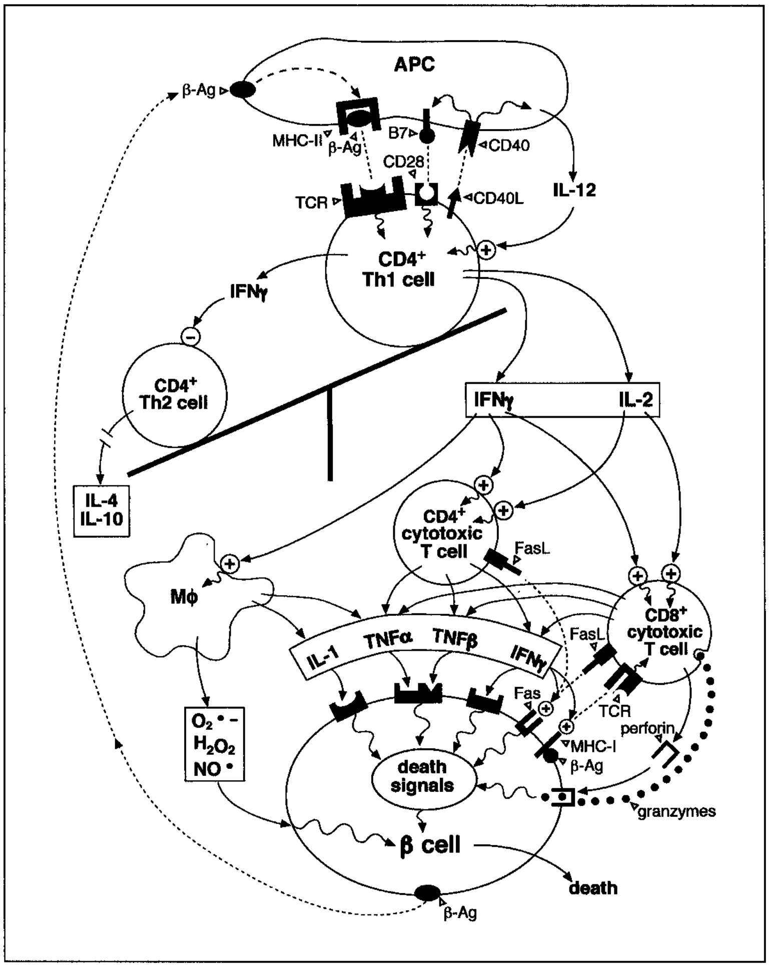

FIGURE 2
A scheme of the immune system cells and cytokines believed to mediate destruction of pancreatic islet ß cells in type 1 diabetes. The concept illustrated posits that certain ß cell protein(s) are processed by antigen-presenting cells (APC), such as macrophages and dendritic cells, then presented as antigen(s) (ß-Ag) in a complex with MHC class II molecules on the surface of the APC. APC and CD4+ T cells interact via i) the binding of a ß-Ag-MHC II complex on the APC surface to a T cell receptor (TCR) specific for ß-Ag, ii) the binding of costimulator molecules (e.g., B7, CD40, ICAM-1) on the APC surface to their corresponding receptors or ligands (e.g., CD28, CD40L, LFA- 1) on the T cell, and iii) the production by the APC of cytokines such as IL-12 that promote differentiation of CD4+ T cells into Th1-type cells. Collectively, these interactions, and perhaps others, activate CD4+ Th1 cells to produce their characteristic cytokines (IFNγ, IL-2). IFNγ i) inhibits CD4+ Th2 cell production of IL-4 and IL-10, and ii) activates macrophages (Mϕ) and cytotoxic T cells; also, IL-2 activates cytotoxic T cells. CD8+ T cells are cytotoxic to ß cells following specific recognition of ß-Ag on the ß cell. This necessitates direct contact of CD8+ T cells with ß cells via the binding of a CD8+ TCR specific for ß-Ag to the ß-Ag-MHC I complex on the ß cell surface. This T cell-ß cell interaction activates CD8+ T cells, and these cells may then destroy ß cells via i) the binding of Fas ligand (FasL) on the CD8+ T cell to a Fas receptor on the ß cell, and ii) the secretion of cytotoxic molecules, such as perforin and granzymes. In addition, T cells and Mϕ may destroy ß cells indirectly, that is, the immunologic cells are not in direct contact with ß cells and there is no requirement for specific recognition of ß-Ag on ß cells. Rather, activated Mϕ may destroy ß cells by producing free radicals, such as superoxide ($O_2^{\bullet -}$), hydrogen peroxide ($H_2O_2$), and nitric oxide ($NO^{\bullet}$), and cytokines (IL-1, TNFα) that are cytotoxic to ß cells. Also, activated CD4+ T cells and CD8+ T cells may destroy ß cells by producing cytokines (TNFα, TNFß, IFNγ) that are cytotoxic to ß cells. In addition, cytokines (IL-1, TNFα, TNFß, IFNγ) may i) induce Fas receptors on ß cells and so allow CD4+ and CD8+ T cells to destroy the ß cells via FasL/Fas-mediated mechanisms, and ii) increase expression of MHC-I molecules on ß cells and so increase interactions of CD8+ T cells and ß cells. Finally, ß cell death may result from direct toxic effects of free radicals (death by necrosis), and from actions of cytokines (IL-1, TNFα, TNFß, IFNγ), FasL/Fas, perforin and granzymes that activate death signals (e.g., caspase enzymes) in ß cells and lead to ß cell self- destruction (death by apoptosis and sometimes necrosis). (Reproduced from Rabinovitch A. Roles of cell-mediated immunity and cytokines in the pathogenesis of Type 1 diabetes mellitus: In *Diabetes Mellitus: A Fundamental and Clinical Text*. 2nd edition, 2000, Eds. LeRoith, Taylor, Olefsky, with permission of Lippincott Williams & Wilkins.)

cells through a variety of antigen-specific and nonspecific mechanisms (Fig. 2).

### Immunostimulatory Procedures to Prevent Type 1 Diabetes

The concept has been presented above that the autoimmune response in type 1 diabetes involves disturbances in immunoregulatory circuits that may be manifested as dominance of Th1 over Th2 cell function and cytokine production (Fig. 2). A corollary of this proposition is that measures leading to reversal of this Th subset balance, with Th2 cells/cytokines dominating over Th1 cells/cytokines, should block the autoimmune response and prevent diabetes development. There is evidence to support this hypothesis. Thus, administrations of a variety of "immunostimulants" — microbial agents, immune adjuvants, and T cell mitogens — have been discovered to prevent the development of insulitis, ß cell destruction, and autoimmune diabetes in genetically diabetes-prone NOD mice and BB rats.[79-101] Importantly, these immunostimulatory procedures prevented diabetes development without structural changes or complete remodel-

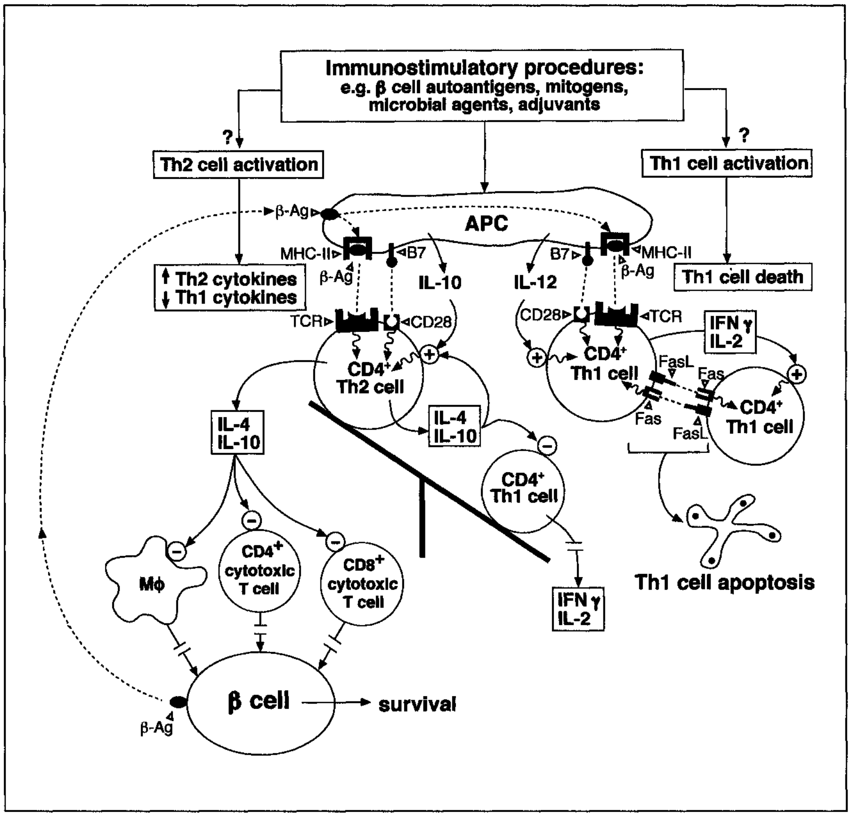

FIGURE 3
Two distinct mechanisms by which immunostimulatory procedures (e.g., ß cell autoantigens, mitogens, microbial agents, adjuvants), possibly acting via APC stimulation, may prevent or block the autoimmune response leading to ß cell destruction in type 1 diabetes. One mechanism may be by Th2 cell activation. Thus, strong B7-CD28 costimulation during APC-CD4+ T cell interactions is thought to favor differentiation of Th2 over Th1 cells. Also, IL-4 and IL-10 induce Th2 over Th1 cell differentiation. Th2 cells produce IL-4 and IL-10 which downregulate Th1 cells that produce IFNγ and IL-2. The combination of increased IL-4 and IL-10 production and decreased IFNγ and IL-2 production inhibits cytotoxic Mϕ and T cell activities, thereby preventing ß cell damage and diabetes development. A second mechanism by which immunostimulatory procedures may prevent autoimmune ß cell destruction may be by activating ß cell-autoreactive Th1 cells along pathways leading to their self-destruction (apoptosis) by IFNγ and IL-2-dependent, FasL/Fas-mediated mechanisms, while Th2 cells that are relatively resistant to activation-induced cell death would survive. (Reproduced from Rabinovitch A. Roles of cell-mediated immunity and cytokines in the pathogenesis of Type 1 diabetes mellitus: In *Diabetes Mellitus: A Fundamental and Clinical Text.* 2nd edition, 2000. Eds. LeRoith, Taylor, Olefsky, with permission of Lippincott Williams & Wilkins).

ling of the immune system — unlike procedures that involve bone marrow, thymic, or lymphoid cell replacement or deletion (e.g., anti-lymphocyte serum, cyclosporine, monoclonal antibodies to T cells, silica, and anti-macrophage antibodies).[8] Rather, the diabetes-preventive effects of immune adjuvants have been attributed to stimulation of T regulatory (suppressor) cells and cytokines whose effects were to suppress[95-98] or render dormant[94] autoreactive T cells. Taken together, these studies suggest that certain immunostimulatory procedures may reset the Th subset balance so that Th2 cells/cytokines dominate over Th1 cells/cytokines (Fig. 3).

The hypothesis that immunostimulatory procedures may prevent diabetes development in autoimmune diabetes-prone rodents by upregulating Th2 cells/cytokines is supported by several lines of evidence. Complete Freund's adjuvant (CFA)-induced protection of NOD mice from ß cell- destructive insulitis and diabetes was found to be associated with a relative increase in IL-4-producing cells and a decrease in IFNγ-producing cells recovered from "sentinel" syngeneic islet grafts placed under the renal capsule.[100] However, in a subsequent study, it was found that diabetes suppression following CFA administration to diabetes-prone NOD mice may be mediated only in part by Th2-type cytokines because combined anti-IL-4 and anti-IL-10 antibody treatment induced a state of glucose intolerance but did not abrogate diabetes prevention by CFA.[101] In another study, treatment of already diabetic NOD mice with CFA at the time of syngeneic islet transplantation prevented destruction of ß cells in the islet graft and diabetes did not recur.[93] Lymphocytes and monocytes/macrophages still accumulated around the transplanted islets (peri-islet insulitis) in the CFA-treated NOD mice, but these mononuclear cells did not invade the islets and ß cells remained intact.[93] In yet another study, IL-10 mRNA expression was significantly increased and IL-2 and IFNγ mRNA levels were significantly decreased in syngeneic islet grafts of CFA-injected NOD mice compared with saline-injected NOD mice.[102] This suggested that CFA treatment upregulated IL-10 production in the islet graft, resulting in decreased production of Th1 cytokines (IL-2 and IFNγ) and conversion of a ß cell-destructive islet infiltrate into a nondestructive peri-insulitis lesion. This interpretation was supported in a subsequent study, in which the combined administration of IL-10 plus IL-4 (Th2 cytokines) was found to produce significantly prolonged survival of syngeneic islet grafts in diabetic NOD mice.[103]

The diabetes-preventive effects of IL-4 and IL-10 are in accord with the known actions of these cytokines to downregulate inflammatory responses mediated by monocytes/macrophages and their cytokine products, as well as to downregulate cell-mediated immune responses triggered by Th1 cells and their cytokine products.[34,37] Indeed, IL-4 is consistently diabetes-preventive in NOD mice, either expressed transgenically by ß cells,[104] or administered systemically.[51] Transgenic studies suggest a proinflammatory and diabetogenic role for IL-10 when this cytokine is expressed locally in islets;[105-107] however, systemic administrations of IL-10[108,109] and islet-specific T cells that hyperexpress IL-10 (by gene transfection)[110] have been reported to prevent diabetes development in NOD mice.

Interestingly, the ability of immunostimulatory procedures, such as microbial agents and immune adjuvants to promote Th2 over Th1 immune responses is concordant with the concept that the intensity of T cell signalling can dramatically affect the balance of Th1/Th2 subsets. According to this "strength of signal" hypothesis, any reagent or situation that results in strong costimulation of CD28 receptors on T cells by B7 costimulatory molecules on APCs will promote Th2 immune responses, whereas lower intensities of B7/CD28 costimulation will promote Th1 responses.[111] In support of this hypothesis, diabetes in NOD mice is *exacerbated* when the mice are bred onto the CD28 knockout background as a direct result of a reduction in the protective Th2 response and concomitant enhancement of the Th1 response.[112] Also, activation of CD28 signalling in T cells by anti-CD28 monoclonal antibody treatment of NOD mice at 2 weeks of age (but not at 5-6 weeks) was recently reported to increase IL-4 production by islet-infiltrating T cells and prevent diabetes development.[113] These findings suggest that immunostimulatory procedures may promote Th2 immune responses and prevent diabetes by upregulating B7/CD28 costimulation (Fig. 3). Recently, it was reported that B7-1 and B7-2 expression is decreased on dendritic cells in peripheral blood of humans at high risk for type 1 diabetes, and this was accompanied by reduced stimulation of autologous CD4+ T cells.[114] Therefore, according to the strength of signal hypothesis, *low* levels of B7/CD28 costimulation in individuals at risk for type 1 diabetes would favor a Th1 cell-mediated immune response that destroys islet ß cells at the expense of a protective Th2 response.

Recent studies suggest a novel mechanism for differential regulation of Th1 and Th2 subsets, namely a differential ability of Th1 and Th2 cells to undergo activation-induced cell death (AICD), also termed apoptosis. Thus, Th1, but not Th2, cells have been reported to undergo rapid FasL/Fas-mediated apoptosis after antigen stimulation.[115-117] Therefore, it is tempting to speculate that immunostimulatory procedures, such as microbial agents, adjuvants, mitogens, and ß cell autoantigens, might prevent autoimmune diabetes development by preferentially inducing apoptosis of autoreactive Th1-type cells (Fig. 3). According to this scenario, prevention of autoimmune destruction of ß cells would be associated with a decrease in the ratio of Th1/Th2 cells as a consequence of decreases in Th1 cells *without* any increase in Th2 cells, rather their selective survival.

Indeed, this has recently been reported to be the mechanism of the protective effect of immune adjuvants against diabetes development in NOD mice. It was found that BCG and CFA-induced diabetes prevention in NOD mice *persisted* in NOD mice genetically deficient in either IL-4 or IL-10, whereas IFNγ-deficient NOD mice were *not* protected from diabetes by BCG or CFA.[118] Thus, immune adjuvants protected against diabetes by mechanisms *independent* of Th2-type cytokines (IL-4 and IL-10); rather, the Th1-type cytokine, IFNγ was required, unexpectedly, for immune adjuvant- induced diabetes prevention. The dependency on IFNγ for immune adjuvant-induced diabetes prevention was due, presumably, to deletion of autoreactive Th1 cells by IFNγ, because NOD Th1 splenic cells were more sensitive to activation-induced cell death than NOD Th2 splenic cells.[118] Similarly, IFNγ, induced by BCG infection of nondiabetes-prone mice, has been reported to act as regulator of the immune response by inducing apoptosis of CD4 T cells initially activated by BCG.[119]

Anti-T cell antibodies have been found to induce tolerance and prevent ß cell destruction in NOD mice, even when the antibodies are administered after insulitis has started and effector T cells have been activated.[120] The mechanism of nondepleting anti-CD4 monoclonal antibody to induce tolerance in a primed immune system has been reported to be by activation of CD4+ T regulatory cells,[121] and recently by direct prevention of effector cell function, presumably by deletion of activated autoreactive T cells.[122] Other studies have revealed that induction of tolerance to cardiac and pancreatic islet allografts in mice is critically dependent upon IFNγ[123,124] and IL-2[125,126] production. This supports the concept that Th1 cell activation can lead to self-deletion via apoptosis and, consequently, specific T cell tolerance to the stimulating antigen.

In addition, the protective effect of peripheral NKT cells against autoimmunity in NOD mice, originally proposed to be due to shifting the profile of autoreactive T cells toward a protective Th2 type,[127] was recently reported to be related, instead, to IL-12-induced activation and IFNγ secretion by NKT cells, and these Th1 type immunoregulatory responses were deficient in NOD mice.[128] Further evidence that Th1 cell activation is required to prevent autoimmune diabetes development was provided by a recent study that reported acceleration of diabetes in NOD mice in which endogenous IL-12 was neutralized by anti-IL-12 antibody administered to young NOD mice (2 weeks of age) for 6 days only.[129] By contrast, when anti-IL-12 antibody was administered to older NOD mice (from age 5 to 30 weeks), insulitis and diabetes were suppressed.[129] These findings reveal the dual role of Th1 cytokines (IL-12 and IFNγ): i) they act as early *regulators* of immune responses, by deleting autoreactive Th1 cells and, if this regulatory action is inadequate and islet ß cell autoreactive Th1 cells persist, then ii) they act as *effectors* of ß cell destruction.

The aforementioned studies support the general consensus that Th1 cells/cytokines are the major disease effectors in autoimmune diabetes,[130-133] and that deletion of Th1 cells or blockade of Th1 cell/cytokine actions can prevent diabetes development. There is conflicting evidence, however, on whether Th2 cells/cytokines have a protective effect. For example, cotransfer of polarized Th1 and Th2 cells did not inhibit the ability of the Th1 population to provoke diabetes.[47] Also, NOD mice with an IL-4 gene knockout mutation did not manifest intensified insulitis or accelerated diabetes.[134] These findings do not support the concept that Th2 cells provide dominant protection against ß cell destruction in the insulitis lesion. This conclusion must be tempered, though, by the fact that IL-4 knockout mice still produce other Th2 cell-derived cytokines (e.g., IL-5, IL-10),[135,136] and possibly the Th3 cell-derived cytokine, TGFß, any or all of which could still downregulate Th1 cytokine production in IL-4-deficient NOD mice.

### Future Prospects: Clinical Considerations

The clinical hope from observations that certain immunostimulatory procedures prevent autoimmune diabetes development in genetically diabetes-prone animals is that clinically safe means of immune stimulation may be similarly effective in preventing type 1 diabetes in human subjects at risk for this disease. Immunostimulatory agents that have a broad spectrum of immune stimulation affecting macrophages and T cells (e.g., the immune adjuvant, bacille Calmette-Guérin [BCG] vaccine) and polyclonal T cell activators (e.g., microbial superantigens, lectins) may not be optimal for clinical trials because of possible undesirable side effects from generalized immunostimulation.

Recent findings, however, demonstrate that more selective immunostimulation may be at hand. Thus; administration of the peptide GAD65, an islet ß cell autoantigen, can prevent autoimmune diabetes development in NOD mice, and this prevention is associated with the induction of specific tolerance to this peptide.[137,138] Moreover, GAD-responsive T cells from diabetes-prone NOD mice were characterized as Th1, IFNγ-producing.[137] In contrast, IFNγ production was reduced in antigen-stimulated spleen cell cultures from GAD65-tolerant (and diabetes-protected) NOD mice, indicating that tolerance may result from suppression of GAD65-responsive Th1 cells.[138] Because this effect was not accompanied by a corresponding reduction of the humoral (antibody) response to GAD and other ß cell autoantigens, a GAD65 induction of Th2 cells with suppression of Th1 cells was suggested.[138] Importantly, GAD65 administration to NOD mice was reported to suppress an *ongoing diabetogenic response* (late insulitis, prehyperglycemic stage of type 1 diabetes), and this protection was mediated through the induction of regulatory $CD4^+$ T cells with a Th2 phenotype.[139] Furthermore, induction of GAD65-specific Th2 cells and suppression of diabetes in NOD mice is IL-4 dependent, because NOD mice genetically deficient in IL-4 production (IL-4 gene knockout NOD mice) were *not* protected from diabetes development after immunization with GAD65-specific peptides,[140] or a novel plasmid DNA construct encoding both a GAD65 peptide linked to IgG Fc and IL-4.[141] These findings are directly relevant to reports that there is an inverse relation between humoral (Th2 cell-mediated) and cellular (Th1 cell-mediated) autoimmunity to GAD in human subjects at risk for type 1 diabetes[142] and that a strong humoral (serum antibody) response to GAD correlates with a slow progression to diabetes.[142,143]

Administration of ß cell candidate autoantigens other than GAD may also induce self- tolerance and prevent diabetes development. For example, insulin (and insulin B chain) can prevent diabetes in NOD mice and BB rats, and possibly in human subjects at high risk for type 1 diabetes.[144] Recently, a T cell response to a particular epitope of the insulin B chain, $B_{(9-23)}$ was described in peripheral blood lymphocytes obtained from human subjects with recent-onset type 1 diabetes and from prediabetic subjects at high risk for disease, and these T cells produced IFNγ.[145] The significance of these findings is that therapies that are directed at this autoantigenic response might be of benefit in controlling human type 1 diabetes, as was achieved by administration of the B chain or $B_{(10-24)}$ peptide of insulin in NOD mice.[146,147] In addition, reports that NOD mice can be protected from diabetes development by administering the ß cell autoantigens, GAD[148,149] and insulin[146,147,150-154] by oral, intranasal or aerosol inhalation routes may be of practical importance for clinical application. The mechanisms of the protective effects of these treatments in NOD mice have been ascribed to activation of $CD4^+$ αß T cells or $CD8^+$ γδ T cells that produced one or more suppressor cytokines (IL-4, IL-10 and TGFß).

Immune-mediated destruction of insulin-secreting ß cells precedes the overt expression of clinical symptoms by many years because these become apparent only when a majority of the ß cells have been destroyed. Interrupting this pathogenetic sequence by immune intervention offers the opportunity to alter the natural history of type 1 diabetes. Several approaches are currently being explored in clinical trials or are under consideration for such trials. These include the following therapeutic approaches used singly or in combination: i) administration of ß cell autoantigens (e.g., insulin) via parenteral, oral, nasal or aerosol inhalation routes; ii) manipulation of expression of costimulatory molecules (e.g., B7/CD28, CD40/CD40L) on antigen-presenting cells and T cells in attempts to delete autoreactive Th1 cells or direct T cell signalling pathways from Th1 to Th2 cell dominance; and iii) administration of cytokine-based therapies (e.g., cytokines, antibodies to cytokines and cytokine receptors, soluble cytokine receptors and receptor antagonists, cytokine receptor-targeted cytotoxic drugs) to block the production and/or action of proinflammatory cytokines (IL-1 and TNFα) and type 1 cytokines (IFNγ, IL-2, TNFß and IL-12), while maintaining or increasing the production and/or action of regulatory cytokines (IL-4, IL-10, TGFß).

### Acknowledgements

The authors thank Chris Bleackley, Tim Mosmann, Robert Power, Jonathan Lakey, Ray Rajotte, David Serreze, Bhagirath Singh, Hui-Yu Qin and Michel Sadelain who have contributed to the research from their laboratory cited in this review. This work was supported by the Alberta Heritage Foundation for Medical Research, the Canadian Institutes for Health Research, the Juvenile Diabetes Foundation International, the Canadian Diabetes Association, the Muttart Diabetes Research and Training Centre at the University of Alberta, and the MacLachlan Fund of the University of Alberta Hospitals.

### References

[1] Bach, J. F. (1994). Insulin-dependent diabetes mellitus as an autoimmune disease, *Endocrine Rev*, 15, 516 - 542.

[2] Wilkin, T. J. (1990). The primary lesion theory of autoimmunity: a speculative hypothesis, *Autoimmunity*, 7, 225 - 235.

[3] Lampeter, E. F., Homberg, M., Quabeck, K. Schaefer V. W., Wernet, P., Bertrams, J., Grosse-Wilde, H., Gries, F. A. and Kolb, H. (1993). Transfer of insulin-dependent diabetes between HLA-identical siblings by bone marrow transplantation, *Lancet*, 341, 1243 - 1244.

[4] Vialettes, B., Maraninchi, D., San Marco, M. P., Birg, F., Stoppa, A. M., Mattei-Zevaco, C., Thivolet, C., Hermitte, L., Vague, P. and Mercier, P. (1993). Autoimmune polyendocrine failure—Type 1 (insulin-dependent) diabetes mellitus and hypothyroidism—after allogeneic bone marrow transplantation in a patient with lymphoblastic leukaemia, *Diabetologia*, 36, 541 - 546.

[5] Lampeter, E. B. (1993). Discussion remark to Session 24: BMT in autoimmune diseases, *Exp Hematol*, 21, 1155.

[6] Calcinaro, F., Hao, L., Chase, H. P., Klingensmith, G. and Lafferty, K. J. (1992). Detection of cell-mediated immunity in type I diabetes mellitus, *J Autoimmun*, 5, 137 - 147.

[7] Petersen, J. S., Marshall, M. O., BÊkkeskov, S., Hejnaes, K. R., Hoier-Madsen, M. and Dyrberg, T. (1993). Transfer of Type 1 (insulin-dependent) diabetes mellitus associated autoimmunity to mice with severe combined immunodeficiency (SCID), *Diabetologia*, 36, 510 - 515.

[8] Rossini, A. A., Greiner, D. L., Friedman, H. P. and Mordes, J. P. (1993). Immunopathogenesis of diabetes mellitus, *Diabetes Rev*, 1, 43 - 75.

[9] Singh, B. and Rabinovitch, A. (1993). Influence of microbial agents on the development and prevention of autoimmune diabetes, *Autoimmunity*, 15, 209 - 213.

[10] Dardenne, M., Lepault, F., Bendelac, A. and Bach J. F. (1989). Acceleration of the onset of diabetes in NOD mice by thymectomy at weaning, *Eur J Immunol*, 19, 889 - 895.

[11] Harada, M. and Makino, S. (1984) Promotion of spontaneous diabetes in non-obese diabetes-prone mice by cyclophosphamide, *Diabetologia*, 27, 604 - 606.

[12] Yasunami, R. and Bach, J. F. (1988). Anti-suppressor effect of cyclophosphamide on the development of spontaneous diabetes in NOD mice, *Eur J Immunol*, 18, 481 - 484.

[13] Bendelac, A., Carnaud, C., Boitard, C. and Bach J. F. (1987). Syngeneic transfer of autoimmune diabetes from diabetic NOD mice to healthy neonates. Requirement for both L3T4+ and Lyt-2+ T cells, *J Exp Med*, 166, 823 - 832.

[14] Haskins, K. and McDuffie, M. (1990). Acceleration of diabetes in young NOD mice with a CD4+ islet-specific T cell clone, *Science*, 249, 1433 - 1436.

[15] Sempé, P., Richard, M. F., Bach, J. F. and Boitard, C. (1994). Evidence of CD4+ regulatory T cells in the nonobese diabetic male mouse, *Diabetologia*, 37, 337 - 343.

[16] Boitard, C., Yasunami, R., Dardenne, M. and Bach, J. F. (1989). T cell-mediated inhibition of the transfer of autoimmune diabetes in

NOD mice, *J Exp Med*, **169**, 1669 - 1680.

[17] Pankewycz, O. G., Guan, J. X. and Benedict, J. F. (1992). A protective NOD islet-infiltrating CD8+ T cell clone, I.W. 2.15, has *in vitro* immunosuppressive properties, *Eur J Immunol*, **22**, 2017 - 2023.

[18] Pankewycz, O., Strom, T. B., Rubin-Kelley, V. E. (1991). Islet-infiltrating T cell clones from non-obese diabetic mice that promote or prevent accelerated onset diabetes, *Eur J Immunol*, **21**, 873 - 879.

[19] Diaz-Gallo, C., Moscovitch-Lopatin, M., Strom, T. B. and Kelley, V. R. (1992). An anergic, islet-infiltrating T-cell clone that suppresses murine diabetes secretes a factor that blocks interleukin 2/interleukin 4-dependent proliferation, *Proc Natl Acad Sci USA*, **89**, 8656 - 8660.

[20] Boitard, C., Bendelac, A., Richard, M. F., Carnaud, C. and Bach, J. F. (1988). Prevention of diabetes in nonobese diabetic mice by anti-I-A monoclonal antibodies: transfer of protection by splenic T cells, *Proc Natl Acad Sci USA*, **85**, 9719 - 9723.

[21] Greiner, D. L., Mordes, J. P., Handler, E. S., Angelillo, M., Nakamura, N, and Rossini, A. A. (1987). Depletion of RT6.1+ T lymphocytes induces diabetes in resistant biobreeding/Worcester (BB/W) rats, *J Exp Med*, **166**, 461 - 475.

[22] Rossini, A. A., Faustman, D., Woda, B. A., Like, A. A., Szymanski, I. and Mordes, J.P. (1984). Lymphocyte transfusions prevent diabetes in the Bio-Breeding/Worcester rat, *J Clin Invest*, **74**, 39 - 46.

[23] Thorpe, R., Wadhwa, M., Bird, C. R. and Mire-Sluis, A. R. (1992). Detection and measurement of cytokines, *Blood Rev*, **6**, 133 - 148.

[24] Mosmann, T. R., Cherwinski, H., Bond, M. W., Giedlin, M. A. and Coffman, R. L. (1986). Two types of murine helper T cell clone: I. Definition according to profiles of lymphokine activities and secreted proteins, *J Immunol*, **136**, 2348 - 2357.

[25] Cherwinski, H. C., Schumacher, J. H., Brown, K. D. and Mosmann, T. R. (1987). Two types of mouse helper T cell clone. III. Further differences in lymphokine synthesis between Th1 and Th2 clones revealed by RNA hybridization, functionally monospecific bioassays and monoclonal antibodies, *J Exp Med*, **166**, 1229 - 1244.

[26] Del Prete, G. F., De Carli, M., Mastromauro, C., Biagiotti, R., Macchia, D., Falagiani, P., Ricci, M. and Romagnani, S. (1991). Purified protein derivative (PPD) of *Mycobacterium tuberculosis* and excretory-secretory antigen(s) (TES) of *Toxocara canis* expand in vitro human T cells with stable and opposite (type 1 T helper or type 2 T helper) profiles of cytokine production, *J Clin Invest*, **88** 346 - 350.

[27] Mosmann, T. R. and Sad, S. (1996) The expanding universe of T-cell subsets: Th1, Th2 and more, *Immunol Today*, **17**, 138 - 146.

[28] Mosmann, T. R. and Coffman, R. L. (1989). TH1 and TH2 cells: different patterns of lymphokine secretion lead to different functional properties, *Annu Rev Immunol*, **7**, 145 - 173.

[29] Chen, Y., Kuchroo, V. K., Inobe, J., Hafler, D. A. and Weiner, H. L. (1994). Regulatory T cell clones induced by oral tolerance: suppression of autoimmune encephalomyelitis, *Science*, **265**, 1237 - 1240.

[30] Romagnani, S. (1994). Lymphokine production by human T cells in disease states, *Annu Rev Immunol*, **12**, 227 - 257.

[31] Fitch, F. W., McKisic, M. D., Lancki, D. W. and Gajewski, T. F. (1993). Differential regulation of murine T lymphocyte subsets, *Annu Rev Immunol*, **11**, 29 - 48.

[32] Seder, R. A. and Paul, W. E. (1994). Acquisition of lymphokine-producing phenotype by CD4+ T-cells, *Annu Rev Immunol*, **12**, 635 - 673.

[33] Moore, K. W., O'Garra, A., de Waal Malefyt, R., Vieira, P. and Mosmann, T. R. (1993). Interleukin 10, *Annu Rev Immunol*, **11**, 165 - 190.

[34] Trinchieri, G. (1995). Interleukin-12: a proinflammatory cytokine with immunoregulatory functions that bridge innate resistance and antigen-specific adaptive immunity, *Annu Rev Immunol*, **13**, 251 - 276.

[35] Ding, L., Linsley, P. S., Huang, L-Y., Germain, R. N. and Shevach, E. M. (1993). IL-10 inhibits macrophage costimulatory activity by selectively inhibiting the upregulation of B7 expression, *J Immunol*, **151**, 1224 - 1234.

[36] Powrie, F., Menon, S., Coffman, R. L. (1993). Interleukin-4 and interleukin-10 synergize to inhibit cell-mediated immunity in vivo, *Eur J Immunol*, **23**, 3043 - 3049.

[37] Mosmann, T. (2000). Complexity or coherence? Cytokine secretion by B cells, *Nature Immunol*, **1**, 465 - 466.

[38] Clerici, M., Shearer, G. M. (1994). The Th1-Th2 hypothesis of HIV infection: new insights, *Immunol Today*, **15**, 575 - 581.

[39] Fowell, D., McKnight, A. J., Powrie, F., Dyke, R. and Mason, D. (1991). Subsets of CD4+ T-cells and their roles in the induction and prevention of autoimmunity, *Immunol Rev*, **123**, 37 - 64.

[40] Fowell, D. and Mason, D. (1993). Evidence that the T cell repertoire of normal rats contains cells with the potential to cause diabetes. Characterization of the CD4+ T cell subset that inhibits this autoimmune potential, *J Exp Med*, **177**, 627 - 636.

[41] Shimada, A., Rohane, P., Fathman, C. G. and Charlton, B. (1996). Pathogenic and protective roles of $CD45RB^{low}$ $CD4^{+}$ cells correlate with cytokine profiles in the spontaneously autoimmune diabetic mouse, *Diabetes*, **45**, 71 - 78.

[42] Zekzer, D., Wong, F. S., Ayalon, O., Millet, I., Altieri, M., Shintani, S., Solimena, M. and Sherwin, R. S. (1998). GAD-reactive CD4+ Th1 cells induce diabetes in NOD/SCID mice, *J Clin Invest* **101**, 68 - 73.

[43] Akhtar, I., Gold, J. P., Pan, L-Y., Ferrara, J. L., Yang, X. D., Kim, J. I. and Tan, K. N. (1995). $CD4^{+}$ ß islet cell-reactive T cell clones that suppress autoimmune diabetes in nonobese diabetic mice, *J Exp Med*, **182**, 87 - 97.

[44] Han, H-S., Jun, H-S., Utsugi, T. and Yoon, J. W. (1996). A new type of CD4+ suppressor T cell completely prevents spontaneous autoimmune diabetes and recurrent diabetes in syngeneic islet-transplanted NOD mice, *J Autoimmun* **9**, 331 - 339.

[45] Zekzer, D., Wong, F. S., Wen, L., Altieri, M., Gurlo, T, von Grafenstein, H. and Sherwin, S. (1997). Inhibition of diabetes by an insulin-reactive CD4 T-cell clone in the nonobese diabetic mouse, *Diabetes*, **46**, 1124 - 1132.

[46] Healey, D., Ozegbe, P., Arden, S., Chandler, P., Hutton, J. and Cooke, A. (1995). *In vivo* activity and *in vitro* specificity of CD4+ Th1 and Th2 T cells derived from the spleens of diabetic NOD mice, *J Clin Invest*, **95**, 2979 - 2985.

[47] Katz, J. D., Benoist, C. and Mathis, D. (1995). T helper cell subsets in insulin-dependent diabetes, *Science*, **268**, 1185 - 1188.

[48] Hammond, K. J. L., Poulton, L. D., Palmisano, L. J., Silveira, P. A., Godfrey, D. I. and Baxter, A. G. (1998). αß/-T cell receptor $(TCR)^{+}$CD4CD8 (NKT) thymocytes prevent insulin-dependent diabetes mellitus in nonobese diabetic (NOD)/Lt mice by the influence of interleukin (IL)-4 and/or IL-10, *J Exp Med*, **187**, 1047 - 1056.

[49] Gombert, J-M., Herbelin, A., Tancrede-Bohin, E., Dy, M., Carnaud, C. and Bach, JF (1996). Early quantitative and functional deficiency of $NK1^{+}$-like thymocytes in the NOD mouse, *Eur J Immunol*, **26**, 2989 - 2998.

[50] Godfrey, D. I., Kinder, S. J., Silveira, P. and Baxter, A. G. (1997). Flow cytometric study of T cell development in NOD mice reveals a deficiency in $/TCR^{+}$CD4CD8 thymocytes, *J Autoimmun*, **10**, 279 - 285.

[51] Rapoport, M. J., Jaramillo, A., Zipris, D., Lazarus, A. H., Serreze, D. V., Leiter, E. H., Cyopick, P., Danska, J. S. and Delovitch, T. L.

(1993). Interleukin-4 reverses T cell proliferative unresponsiveness and prevents the onset of diabetes in nonobese diabetic mice, *J Exp Med*, **178**, 87 - 99.

[52] Herbelin, A., Gombert, J-M., Lepault, F., Bach, J. F and Chatenoud, L. (1998). Mature mainstream TCR+CD4+ thymocytes expressing L-selectin mediate "active tolerance" in the nonobese diabetic mouse, *J Immunol*, **161**, 2620 - 2628.

[53] Lee, K., Kim, M. K., Amano, K., Pak, C. Y., Jaworski, M. A., Mehta, J. G. and Yoon, J. W. (1988). Preferential infiltration of macrophages during early stages of insulitis in diabetes-prone BB rats, *Diabetes*, **37**, 1053 - 1058.

[54] Voorbij, H. A., Jeucken, P. H., Kabel, P. J., De Haan, M. and Drexhage, H. A. (1989). Dendritic cells and scavenger macrophages in pancreatic islets of prediabetic BB rats, *Diabetes*, **38**, 1623 - 1629.

[55] Jansen, A., Homo-Delarche, F., Hooijkaas, H., Leenen, P. J., Dardenne, M. and Drexhage, H. A. (1994). Immunohistochemical characterization of monocyte-macrophages and dendritic cells involved in the initiation of insulitis and beta-cell destruction in NOD mice, *Diabetes*, **43**, 667 - 675.

[56] Oschilewski, U., Kiesel, U. and Kolb, H. (1985). Administration of silica prevents diabetes in BB rats, *Diabetes*, **34**, 197 - 199.

[57] Lee, K., Amano, K. and Yoon, J. W. (1988). Evidence for initial involvement of macrophages in development of insulitis in NOD mice, *Diabetes*, **37**, 989 - 991.

[58] Jun, H-S., Yoon, C-S., Zbytnuik, L., van Rooijen, N. and Yoon, J. W. (1999). The role of macrophages in T cell-mediated autoimmune diabetes in nonobese diabetic mice, *J Exp Med*, **189**, 347 - 358.

[59] Jun, H-S., Santamaria, P., Lim, H-W., Zhang, M. L. and Yoon, J. W. (1999). Absolute requirement of macrophages for the development and activation of ß-cell cytotoxic CD8+ T- cells in T-cell receptor transgenic NOD mice, *Diabetes*, **48**, 34 - 42.

[60] Serreze, D. V., Chapman, H. D., Varnum, D. S., Hanson, M. S., Reifsnyder, P. C., Richard, S. D., Fleming, S. A., Leiter, E. H. and Schultz, L. D. (1996). B lymphocytes are essential for the initiation of T cell-mediated autoimmune diabetes: analysis of a new "speed congenic" stock of NOD.Ig mu null mice, *J Exp Med*, **184**, 2049 - 2053.

[61] Noorchashm, H., Noorchashm, N., Kern, J., Rostami, S. Y., Barker, C. F. and Naji, A. (1997). B-cells are required for the initiation of insulitis and sialitis in nonobese diabetic mice, *Diabetes*, **46**, 941 - 946.

[62] Akashi, T., Nagafuchi, S., Anzai, K., Kondo, S., Kitamura, D., Wakana, S., Ono, J., Kikuchi, M., Niho, Y. and Watanabe, T. (1997). Direct evidence for the contribution of B cells to the progression of insulitis and the development of diabetes in non-obese diabetic mice, *Int Immunol*, **9**, 1159 - 1164.

[63] Shimizu J., Kanagawa, O. and Unanue, E. R. (1993). Presentation of -cell antigens to CD4+ and CD8+ T-cells of nonobese diabetic mice, *J Immunol*, **151**, 1723 - 1730.

[64] Nepom, G. T. and Erlich, H. (1991). MHC class-II molecules and autoimmunity, *Annu Rev Immunol*, 9 493 - 525.

[65] Scott, B., Liblau, R., Degermann, S., Marconi, L. A., Ogata, L., Caton, A. J., McDevitt, H. O. and Lo, D. (1994). A role for non-MHC genetic polymorphism in susceptibility to spontaneous autoimmunity, *Immunity*, **1**, 73 - 83.

[66] Singer, S. M., Tisch, R., Yang, X-D., Sytwu, H. K., Liblau, R. and McDevitt, H. O. (1998). Prevention of diabetes in NOD mice by a mutated I-Ab transgene, *Diabetes*, **47**, 1570 - 1577.

[67] van Seventer, G. A., Shimizu, Y. and Shaw, S. (1991). Roles of multiple accessory molecules in T-cell activation, *Curr Opin Immunol*, 3, 294 - 303.

[68] Grewal, I. S. and Flavell, R. A. (1998). CD40 and CD154 in cell-mediated immunity, *Annu Rev Immunol*, **16**, 111 - 135.

[69] Wong, S., Guerder, S., Visintin, I., Reich, E. P., Swenson, K. E., Flavell, R. A. and Janeway C. A. Jr. (1995). Expression of the co-stimulator molecule B7-1 in pancreatic ß-cells accelerates diabetes in the NOD mouse, *Diabetes*, **44** 326 - 329.

[70] Lenschow, D. J., Ho, S. C., Sattar, H., Rhee, L., Gray, G., Nabavi, N., Herald, K. C. and Bluestone, J. A. (1995). Differential effects of anti-B7-1 and anti-B7-2 monoclonal antibody treatment on the development of diabetes in the nonobese diabetic mouse, *J Exp Med*, **181**, 1145 - 1155.

[71] Balasa, B., Krahl, T., Patstone, G., Lee, J., Tisch, R., McDevitt, H. O. and Sarvetnick, N. (1997). CD40 ligand-CD40 interactions are necessary for the initiation of insulitis and diabetes in nonobese diabetic mice, *J Immunol*, **159**, 4620 - 4627.

[72] Yagi, N., Yokono, K., Amano, K., Nagata, M., Tsukamoto, K., Hasegawa, Y, Yoneda, R., Okamoto, N., Moriyama, H., Miki, M., Tominaga, Y., Miyazaki, J., Yagita, H., Okumura, K., Mizoguchi, A., Miki, A., Ide, C., Maeda, S. and Kasuga, M. (1995). Expression of intercellular adhesion molecule 1 on pancreatic beta-cells accelerates beta-cell destruction by cytotoxic T-cells in murine autoimmune diabetes, *Diabetes*, **44**, 744 - 752.

[73] Moriyama, H., Yokono, K., Amano, K., Nagata, M., Hasegawa, Y., Okamoto, N., Tsukamoto, K., Miki, M., Yoneda, R., Yagi, N., Tominaga, Y., Kikutani, H., Hioki, K., Okumura, K., Yagita, H. and Kasuga, M. (1996). Induction of tolerance in murine autoimmune diabetes by transient blockade of leukocyte function-associated antigen-/intercellular adhesion molecule-1 pathway, *J Immunol*, 157, 3737 - 3743.

[74] Martin, S., Heidenthal, E., Schulte, B., Rothe, H. and Kolb, H. (1998). Soluble forms of intercellular adhesion molecule-1 inhibit insulitis and onset of autoimmune diabetes, *Diabetologia*, **41**, 1298 - 1303.

[75] Blotta, M. H., DeKruyff, R. H. and Umetsu, D. T. (1997). Corticosteroids inhibit IL-12 production in human monocytes and enhance their capacity to induce IL-4 synthesis in CD4+ lymphocytes, *J Immunol*, 158, 5589 - 5595.

[76] Trembleau, S., Penna, G., Bosi, E., Mortara, A., Gately, M. K. and Adorini, L. (1995). Interleukin 12 administration induces T helper type 1 cells and accelerates autoimmune diabetes in NOD mice, *J Exp Med*, **181**, 817 - 821.

[77] Hsieh, C-S., Heimberger, A. B., Gold, J. S., O'Garra, A. and Murphy, K. M. (1992). Differential regulation of T helper phenotype development by interleukins 4 and 10 in an αß T-cell-receptor transgenic system, *Proc Natl Acad Sci USA*, **89**, 6065 - 6069.

[78] Seder, R. A., Paul, W. E., Davis, M. M. and Fazekas de St. Groth, B. (1992). The presence of interleukin 4 during in vitro priming determines the lymphokine-producing potential of CD4+ T-cells from T-cell receptor transgenic mice, *J Exp Med*, **176**, 1091 - 1098.

[79] Oldstone, M. B. (1988). Prevention of type I diabetes in nonobese diabetic mice by virus infection, *Science*, **239**, 500 - 502.

[80] Dyrberg, T., Schwimmbeck, P. L. and Oldstone, M. B. (1988). Inhibition of diabetes in BB rats by virus infection, *J Clin Invest*, **81**, 928 - 931.

[81] Wilberz, S., Partke, H. J., Dagnaes-Hansen, F. and Herberg, L. (1991). Persistent MHV (mouse hepatitis virus) infection reduces the incidence of diabetes mellitus in nonobese diabetic mice, *Diabetologia*, **34**, 2 - 5.

[82] Hermitte, L., Vialettes, B., Naquet, P., Atlan, C., Payan, M. J. and Vague, P. (1990). Paradoxical lessening of autoimmune processes in nonobese diabetic mice after infection with the diabetogenic variant of encephalomyocarditis virus, *Eur J Immunol*, **20**, 1297 - 1303.

[83] Takei, I., Asaba, Y., Kasatani, T., Maruyama, T., Watanabe, K.,

Yanagawa, T., Saruta, T. and Ishii, T. (1992). Suppression of development of diabetes in NOD mice by lactate dehydrogenase virus infection, *J Autoimmun*, 5, 665 - 673.

[84] Toyota, T., Satoh, J., Oya, K., Shintani, S. and Okano, T. (1986). Streptococcal preparation (OK-432) inhibits development of type I diabetes in NOD mice, *Diabetes*, 35, 496 - 499.

[85] Satoh, J., Shintani, S., Oya, K., Tanaka, S., Nobunaga, T., Toyota, T. and Goto, Y. (1988). Treatment with streptococcal preparation (OK-432) suppresses anti-islet autoimmunity and prevents diabetes in BB rats, *Diabetes*, 37, 1188 - 1194.

[86] Kawamura, T., Nagata, M., Utsugi, T. and Yoon, J. W. (1993). Prevention of autoimmune type I diabetes by CD4+ suppressor T-cells in superantigen-treated nonobese diabetic mice, *J Immunol*, 151, 4362 - 4370.

[87] Kino, K., Mizumoto, K., Sone, T., Yamaji, T., Watanabe, J., Yamashita, A., Yamaoka, K., Shimizu, K., Ko, K. and Tsunoo, H. (1990). An immunomodulating protein Ling Zhi-8 (LZ-8) prevents insulitis in nonobese diabetic mice, *Diabetologia*, 33, 713 - 718.

[88] Elias, D., Markovits, D., Reshef, T., van der Zee, R. and Cohen, I. R. (1990). Induction and therapy of autoimmune diabetes in the nonobese diabetic (NOD/Lt) mouse by a 65-kDa heat shock protein, *Proc Natl Acad Sci USA*, 87, 1576 - 1580.

[89] Sadelain, M. W. J., Qin, H-Y., Lauzon, J. and Singh, B. (1990). Prevention of type I diabetes in NOD mice by adjuvant immunotherapy, *Diabetes*, 39, 583 - 589.

[90] Sadelain, M. W. J., Qin, H-Y., Sumoski, W., Parfrey, N., Singh, B. and Rabinovitch, A. (1990). Prevention of diabetes in the BB rat by early immunotherapy using Freund's adjuvant, *J Autoimmun*, 3, 671 - 680.

[91] McInerney, M. F., Pek, S. B. and Thomas, D. W. (1991). Prevention of insulitis and diabetes onset by treatment with complete Freund's adjuvant in NOD mice, *Diabetes*, 40, 715 - 725.

[92] Pearce, R. B. and Peterson, C. M. (1991). Studies of concanavalin A in nonobese diabetic mice. I. Prevention of insulin-dependent diabetes, *J Pharmacol Exp Ther*, 258, 710 - 715.

[93] Wang, T., Singh, B., Warnock, G. L. and Rajotte, R. V. (1992). Prevention of recurrence of IDDM in islet-transplanted diabetic NOD mice by adjuvant immunotherapy, *Diabetes*, 41, 114 - 117.

[94] Ulaeto, D., Lacy, P. E., Kipnis, D. M., Kanagawa, O. and Unanue, E. R. (1992). A T-cell dormant state in the autoimmune process of nonobese diabetic mice treated with complete Freund's adjuvant, *Proc Natl Acad Sci USA*, 89, 3927 - 3931.

[95] Qin, H-Y., Suarez, W. L., Parfrey, N., Power, R. F. and Rabinovitch, A. (1992). Mechanisms of complete Freund's adjuvant protection against diabetes in BB rats: induction of non-specific suppressor cells, *Autoimmunity*, 12, 193 - 199.

[96] Qin, H-Y., Sadelain, M. W. Y., Hitchon, C., Lauzon, J. and Singh, B. (1993). Complete Freund's adjuvant-induced T-cells prevent the development and adoptive transfer of diabetes in nonobese diabetic mice, *J Immunol*, 150, 2072 - 2080.

[97] Yagi, H., Matsumoto, M., Suzuki, S., Misaki, R., Suzuki, R., Makino, S. and Harada, M. (1991). Possible mechanism of the preventive effect of BCG against diabetes mellitus in NOD mouse. I. Generation of suppressor macrophages in spleen cells of BCG-vaccinated mice, *Cell Immunol*, 138, 130 - 141.

[98] Yagi, H., Matsumoto, M., Kishimoto, Y., Makino, S. and Harada, M. (1991). Possible mechanism of the preventive effect of BCG against diabetes mellitus in NOD mice. II. Suppression of pathogenesis by macrophage transfer from BCG-vaccinated mice, *Cell Immunol*, 138, 142 - 149.

[99] Lakey, J. R., Singh, B., Warnock, G. L. and Rajotte, R. V. (1994). BCG immunotherapy prevents recurrence of diabetes in islet grafts transplanted into spontaneously diabetic NOD mice, *Transplantation*, 57, 1213 - 1217.

[100] Shehadeh, N. N., LaRosa, F. and Lafferty, K. J. (1993). Altered cytokine activity in adjuvant inhibition of autoimmune diabetes, *J Autoimmun*, 6, 291 - 300.

[101] Calcinaro, F., Gambelunghe, G. and Lafferty, K. J. (1997). Protection from autoimmune diabetes by adjuvant therapy in the non-obese diabetic mouse: The role of interleukin-4 and interleukin-10, *Immunol Cell Biol*, 75, 467 - 471

[102] Rabinovitch, A., Sorensen, O., Suarez-Pinzon, W. L., Power, R. F., Rajotte, R. V. and Bleackley, R. C. (1994). Analysis of cytokine mRNA expression in syngeneic islet grafts of NOD mice: interleukin 2 and interferon gamma mRNA expression correlate with graft rejection and interleukin 10 with graft survival, *Diabetologia*, 37, 833 - 837.

[103] Rabinovitch, A., Suarez-Pinzon, W. L., Sorensen, O., Bleackley, R. C., Power, R. F. and Rajotte, R. V. (1995). Combined therapy with interleukin-4 and interleukin-10 inhibits autoimmune diabetes recurrence in syngeneic islet-transplanted nonobese diabetic mice: analysis of cytokine mRNA expression in the graft, *Transplantation*, 60, 368 - 374.

[104] Mueller, R., Krahl, T. and Sarvetnick, N. (1996). Pancreatic expression of interleukin-4 abrogates insulitis and autoimmune diabetes in nonobese diabetic (NOD) mice, *J Exp Med*, 184, 1093 - 1099.

[105] Wogensen, L., Huang, X. and Sarvetnick, N. (1993). Leukocyte extravasation into the pancreatic tissue in transgenic mice expressing interleukin 10 in the islets of Langerhans, *J Exp Med*, 178, 175 - 185.

[106] Wogensen, L., Lee, M-S. and Sarvetnick, N. (1994). Production of interleukin 10 by islet cells accelerates immune-mediated destruction of ß cells in nonobese diabetic mice, *J Exp Med*, 179, 1379 - 1384.

[107] Moritani, M., Yoshimoto, K., Tashiro, F., Hashimoto, C., Miyazaki, J., Ii, S., Kudo, E., Iwahana, H., Hayashi, Y. and Sano, T. (1994). Transgenic expression of IL-10 in pancreatic islet A cells accelerates autoimmune insulitis and diabetes in non-obese diabetic mice, *Int Immunol*, 6, 1927 - 1936.

[108] Zheng, X. X., Steele, A. W., Hancock, W. W., Stevens, A. C., Nickerson, P. W., Roy- Chaudhury, P., Tian, Y. and Strom, T. B. (1997). A noncytolytic IL-10/Fc fusion protein prevents diabetes, blocks autoimmunity, and promotes suppressor phenomena in NOD mice, *J Immunol*, 158, 4507 - 4513.

[109] Pennline, K. J., Roque-Gaffney, E. and Monahan, M. (1994). Recombinant human IL-10 (rHU IL-10) prevents the onset of diabetes in the nonobese diabetic (NOD) mouse, *Clin Immunol Immunopathol*, 71, 169 - 175.

[110] Moritani, M., Yoshimoto, K., Ii, S., Kondo, M, Iwahana, H., Yamaoka, T., Sano, T., Nakano, N., Kikutani, H. and Itakura, M. (1996). Prevention of adoptively transferred diabetes in nonobese diabetic mice with IL-10-transduced islet-specific Th1 lymphocytes. A gene therapy model for autoimmune diabetes, *J Clin Invest*, 98, 1851 - 1859.

[111] Rulifson, I. C., Sperling, A. I., Fields, P. E., Fitch, F. W. and Bluestone, J. A. (1997). CD28 costimulation promotes the production of Th2 cytokines, *J Immunol*, 158, 658 - 665.

[112] Lenschow, D. J., Rhee, L., Patel, B., Koons, A., Qin, H. Y., Fuchs, E., Singh, B., Thompson, C. B. and Bluestone, J. A. (1996). CD28/B7 regulation of Th1 and Th2 subsets in the development and progression of autoimmune diabetes, *Immunity*, 5, 285 - 293.

[113] Arreaza, G. A., Cameron, M. J., Jaramillo, A., Gill, B. M., Hardy, D., Laupland, K. B., Rapoport, M. J., Zucker, P., Chakrabarti, S., Chensue, S. W., Qin, H. Y., Singh, B. and Delovitch, T. L. (1997). Neonatal activation of CD28 signaling overcomes T cell anergy and prevents autoimmune diabetes by an IL-4 dependent mechanism, *J Clin Invest*, 100, 2243 - 2253.

[114] Takahashi, K., Honeyman, M. C. and Harrison, L. C. (1998). Impaired yield, phenotype, and function of monocyte-derived dendritic cells in humans at risk for insulin-dependent diabetes, *J Immunol*, **161**, 2629 - 2635.

[115] Ramsdell, F., Seaman, M. S., Miller, R. E., Picha, K. S., Kennedy, M. K. and Lynch, D. H. (1994). Differential ability of Th1 and Th2 T cells to express Fas ligand and to undergo activation-induced cell death, *Int Immunol*, **6**, 1545 - 1553.

[116] Varadhachary, A. S., Perdow, S. N., Hu, C., Ramanarayanan, M. and Salgame, P. (1997). Differential ability of T cell subsets to undergo activation-induced cell death, *Proc Natl Acad Sci USA*, **94**, 5778 - 5783.

[117] Zhang, X., Brunner, T., Carter, L., Dutton, R. W., Rogers, P., Bradley, L., Sato, T., Reed, J. C., Green, D. and Swain, S. L. (1997). Unequal death in T helper (Th)1 and Th2 effectors: Th1, but not Th2, effectors undergo rapid Fas/FasL-mediated apoptosis, *J Exp Med*, **185**, 1837 - 1849.

[118] Serreze, D. V., Chapman, H. D., Post, C. M., Johnson, E. A., Suarez-Pinzon, W. L. and Rabinovitch, A. (2001). Th1 to Th2 cytokine shifts in NOD mice: sometimes an outcome, rather than the cause of diabetes resistance elicited by immunostimulation, *J Immunol*, **166**, 1352 - 1359.

[119] Dalton, D. K., Haynes, L., Chu, C-Q., Swain, S. L. and Wittmer, S. (2000). Interferon eliminates responding CD4 T cells during mycobacterial infection by inducing apoptosis of activated CD4 T cells, *J Exp Med*, **192**, 117 - 122.

[120] Parish, N. M., Hutchings, P. R., O'Reilly, L., Quartey-Papafio, R., Healey, D., Ozegbe, P. and Cooke, A. (1995). Tolerance induction as a therapeutic strategy for the control of autoimmune endocrine disease in mouse models, *Immunol Rev*, **144**, 269 - 300.

[121] Waldmann, H. and Cobbold, S. (1998). How do monoclonal antibodies induce tolerance? A role for infectious tolerance? *Annu Rev Immunol*, **16**, 619 - 644.

[122] Phillips, J. M., Harach, S. Z., Parish, N. M., Fehervani, Z., Haskins, K. and Cooke, A. (2000). Nondepleting anti-CD4 has an immediate action on diabetogenic effector cells, halting their destruction of pancreatic ß cells, *J Immunol*, **165**, 1949 - 1955.

[123] Konieczny, B. T., Dai, Z., Elwood, E. T., Saleem, S., Linsley, P. S., Baddoura, F. K., Larsen, C. P., Pearson, T. C. and Lakkis, F. G. (1998). IFN-γ is critical for long-term allograft survival induced by blocking the CD28 and CD40L T cell costimulation pathways, *J Immunol*, **160**, 2059 - 2064.

[124] Diamond, A. and Gill, R. G. (1999). Biphasic roles for IFN in islet allograft immunity and tolerance, *Transplantation*, **67**, S23.

[125] Dai, Z., Konieczny, B. T., Baddoura, F. K. and Lakkis, F. G. (1998). Impaired alloantigen- mediated T cell apoptosis and failure to induce long-term allograft survival in IL-2-deficient mice, *J Immunol*, **161**, 1659 - 1663.

[126] Steiger, J., Nickerson, P. W., Steurer, W., Moscovitch-Lopatin, M. and Strom, T. B. (1995). IL-2 knockout recipient mice reject islet cell allografts, *J Immunol*, **155**, 489 - 498.

[127] Wilson, S. B., Kent, S. C., Patton, K. T., Orban, T., Jackson, R. A., Exley, M., Porcelli, S., Schatz, D. A., Atkinson, M. A., Balk, S. P., Strominger, J. L. and Hafler, D. A. (1998). Extreme Th1 bias of regulatory V24JQ T cells in type 1 diabetes, *Nature*, **391**, 177 - 181.

[128] Falcone, M., Yeung, B., Tucker, L., Rodriguez, E. and Sarvetnick, N. (1999). A defect in interleukin 12-induced activation and interferon γ secretion of peripheral natural killer T cells in nonobese diabetic mice suggests new pathogenic mechanisms for insulin-dependent diabetes mellitus, *J Exp Med*, **190**, 963 - 972.

[129] Fujihira, K., Nagata, M., Moriyama, H., Yasuda, H., Arisawa, K., Nakayama, M., Maeda, S., Kasuga, M., Okumura, K., Yagita, H. and Yokono, K. (2000). Suppression and acceleration of autoimmune diabetes by neutralization of endogenous interleukin-12 in NOD mice, *Diabetes*, **49**, 1998 - 2006.

[130] Rabinovitch, A. (1994). Immunoregulatory and cytokine imbalances in the pathogenesis of IDDM. Therapeutic intervention by immunostimulation? *Diabetes*, **43**, 613 - 621.

[131] Liblau, R. S., Singer, S. M. and McDevitt, H. O. (1995). Th1 and Th2 CD4+ cells in the pathogenesis of organ-specific autoimmune diseases, *Immunol Today*, **16**, 34 - 38.

[132] Charlton, B. and Lafferty, K. J. (1995). The Th1/Th2 balance in autoimmunity. *Curr Opin Immunol*, **7**, 793 - 798.

[133] Delovitch, T. and Singh, B. (1997). The nonobese diabetic mouse as a model of autoimmune diabetes: immune dysregulation gets the NOD, *Immunity*, **7**, 727 - 738.

[134] Wang, B., Gonzalez, A., Höglund, P., Katz, J. D., Benoist, C. and Mathis, D. (1998). Interleukin-4 deficiency does not exacerbate disease in NOD mice, *Diabetes*, **47**, 1207 - 1211.

[135] Pearce, E. J., Cheever, A., Leonard, S., Covalesky, M., Fernandez-Bortran, R., Kohler, G. and Kopf, M. (1996). Schistosoma mansoni in IL-4-deficient mice, *Int Immunol*, **8**, 435 - 444.

[136] Noben-Trauth, N., Kropf, P. and Muller, I. (1996). Susceptibility to *Leishmani major* infection in interleukin-4-deficient mice, *Science*, **271**, 987 - 990.

[137] Kaufman, D. L., Clare-Salzler, M.., Tian, J., Forsthuber, T., Ting, G. S., Robinson, P., Atkinson, M. A., Sercarz, E. E., Tobin, A. J. and Lehmann, P. V. (1993). Spontaneous loss of T-cell tolerance to glutamic acid decarboxylase in murine insulin-dependent diabetes, *Nature*, **366**, 69 - 72.

[138] Tisch, R., Yang, X-D., Singer, S. M., Liblau, R. S., Fugger, L. and McDevitt, H. O. (1993). Immune response to glutamic acid decarboxylase correlates with insulitis in nonobese diabetic mice, *Nature*, **366** 72 - 75.

[139] Tisch, R., Liblau, R. S., Yang, X-D., Liblau, P. and McDevitt, H. O. (1998). Induction of GAD65-specific regulatory T-cells inhibits ongoing autoimmune diabetes in nonobese diabetic mice, *Diabetes*, **47**, 894 - 899.

[140] Tisch, R., Wang, B. and Serreze, D. V. (1999). Induction of GAD65-specific Th2 cells and suppression of autoimmune diabetes at late stages of disease is epitope-dependent, *J Immunol*, **163**, 1178 - 1187.

[141] Tisch, R., Wang, B., Weaver, D. J., Liu, B., Bui, T., Arthos, J. and Serreze, D. V. (2001). Antigen-specific mediated suppression of ß cell autoimmunity by plasmid DNA vaccination, *J Immunol*, **166**, 2122 - 2132.

[142] Harrison, L. C., Honeyman, M. C., DeAizpurua, H. J., Schmidli, R. S., Colman, P. G., Tait, B. D. and Cram, D. S. (1993). Inverse relation between humoral and cellular immunity to glutamic acid decarboxylase in subjects at risk of insulin-dependent diabetes, *Lancet*, **341**, 1365 - 1369.

[143] Yu, L., Gianani, R. and Eisenbarth, G. S. (1994). Quantitation of glutamic acid decarboxylase autoantibody levels in prospectively evaluated relatives of patients with type I diabetes, *Diabetes*, **43**, 1229 - 1233.

[144] Ramiya, V., Muir, A. and Maclaren, N. (1995). Insulin prophylaxis in insulin-dependent diabetes mellitus. Immunological rationale and therapeutic use, *Clin Immunother*, **3**, 177 - 183.

[145] Alleva, D. G., Crowe, P. D., Jin, L., Kwok, W. W., Ling, N., Gottschalk, M., Conlon, P. J., Gottlieb, P. A., Putnam, A. L. and Gaur, A. (2001). A disease-associated cellular immune response in type 1 diabetics to an immunodominant epitope of insulin, *J Clin Invest*, **107**, 173 - 180.

[146] Daniel, D. and Wegmann, D. R. (1996). Protection of nonobese diabetic mice from diabetes by intranasal or subcutaneous administration of insulin peptide B-chain (9-23), *Proc Natl Acad Sci USA*,

93, 956 - 960.

[147] Polanski, M., Melican, N. S., Zhang, J. and Weiner, H. L. (1997). Oral administration of the immunodominant B-chain of insulin reduces diabetes in a co-transfer model of diabetes in the NOD mouse and is associated with a switch from Th1 to Th2 cytokines, *J Autoimmun*, 10, 339 - 346.

[148] Tian, J., Atkinson, M. A., Clare-Salzler, M. C., Herschenfeld, A., Forsthuber, T., Lehmann, P. V. and Kaufman, D. L. (1996). Nasal administration of glutamate decarboxylase (GAD65) peptides induces Th2 responses and prevents murine insulin-dependent diabetes, *J Exp Med*, 183, 1561 - 1567.

[149] Ma, S-W., Zhao, D-L., Yin, Z-Q., Mukherjee, R., Singh, B., Qin, H. Y., Stiller, C. R. and Jevnikar, A. M. (1997). Transgenic plants expressing autoantigens fed to mice to induce oral immune tolerance, *Nat Med*, 3, 793 - 796.

[150] Hancock, W. W., Polanski, M., Zhang, J., Blogg, N. and Weiner, H. L. (1995). Suppression of insulitis in non-obese diabetic (NOD) mice by oral insulin administration is associated with selective expression of interleukin-4 and -10, transforming growth factor-ß, and prostaglandin-E, *Am J Pathol*, 147, 1193 - 1199.

[151] Zhang, Z. J., Davidson, L. E., Eisenbarth, G. and Weiner, H. L. (1991). Suppression of diabetes in NOD mice by oral administration of porcine insulin, *Proc Natl Acad Sci USA*, 88, 10252 - 10256.

[152] Bergerot, I., Fabien, N., Maguer, V. and Thivolet, C. (1994). Oral administration of human insulin to NOD mice generates $CD4^+$ T cells that suppress adoptive transfer of diabetes, *J Autoimmun*, 7, 655 - 663.

[153] Ploix, C., Bergerot, I., Fabien, N., Perche, S., Moulin, V. and Thivolet, C. (1998). Protection against autoimmune diabetes with oral insulin is associated with the presence of IL-4 type 2 T-cells in the pancreas and pancreatic lymph nodes, *Diabetes*, 47, 39 - 44.

[154] Harrison, L. C., Dempsey-Collier, M., Kramer, D. R. and Takahashi, K. (1996). Aerosol insulin induces regulatory CD8 γδ T cells that prevent murine insulin-dependent diabetes, *J Exp Med*, 184 2167 - 2174.

